# The Tuberculous Convalescent

**Published:** 1900-01-20

**Authors:** 


### THE TUBERCULOUS CONVALESCENT.

One of the grave questions which at once confronts those who advocate the open-air treatment of tuberculosis is, What is to be done with the patient when he is cured? In proportion as we realise that the onset of the disease does not depend exclusively upon the virulence of the bacillus but rather upon the lessened resistance of the patient, owing in great measure to the circumstances of his life, and that the curative effect of the open-air or sanatorium method of treatment hinges largely upon the fact that by it the vital powers are restored, and that in the new circumstances of his life the patient is rendered in a manner immune to the effects of the microbe, even though it may for some time still maintain its hold on certain of his tissues—in proportion as we realise this, we see that while such recovery as takes place may be permanent so long as the healthy conditions and surroundings are maintained, it is quite impossible to promise that restoration shall be so complete as to enable the convalescent to withstand a repetition of the evil influences which were the original cause of the disease. What, then, is to be done with these "recovered" but still unstable patients? The sanitarian and the social philosopher will say with one accord, "Alter the conditions of life and labour in our cities; enforce a higher standard of living, and tuberculosis will have no further terrors." We quite agree; just as we look forward with full confidence to the coming of the millenium. But, in the meantime, what we have to deal with is the fact that a large number of these tuberculous patients who, after a comparatively short residence in sanatoria, are quite fitted for a country life, would require a very, very long treatment indeed before they could with confidence be recommended to return to the conditions amid which the disease had originally attacked them. Hence the importance of the suggestion that such patients, instead of returning to their old life, should, wherever such a course seems possible, be encouraged to fix themselves on the land; and, while sacrificing their ambitions, and perhaps, to a large extent, throwing up their chances of a luxurious life, take to the healthy existence of the tiller of the soil and endeavour to scrape together such a livelihood as is possible by small culture—by eggs and butter, poultry, and garden produce. Let there be no illusions. It is not likely that anything done on a small scale will ever again succeed in the face of competition by large concerns; if, that is, we are to measure success by modern standards. The man who has to grind his living out of mother earth must always work hard. Many of us both growl and wonder when we find to how large an extent England is dependent on the foreigner for his farm produce; but if we go to the places where these things come from, we soon see that what is called "small culture" is hard work, entails long hours and frugal living, and gives but little opportunity of putting by. We cannot wonder, then, that the strong, the young, and the ambitious fly from such a life and crowd into towns, where they get a chance of a higher life and a greater reward for their exertions. But this is just what gives the opportunity to those who have once felt the touch of the white fiend, and who know that the light and air of country life is essential to their health even though they be got only by much labour. There is no likelihood that the demand for the products of small husbandry will ever fail. The reward, no doubt, will not be great and not attractive to those who are capable of better work, but to those whose lives depend on light and air, the tilling of the soil—hard work though it be—may be better than a life of comparative ease and comfort in the city, obtained at the cost of the ever-present danger of recurring tuberculosis.

